# Supplementary material for: Integrated deep visual and semantic attractor neural networks predict fMRI pattern-information along the ventral object processing pathway
Source: Sci Rep. 2018 Jul 13;8:10636. doi: 10.1038/s41598-018-28865-1 (PMC6045572; doi:10.1038/s41598-018-28865-1)
Supplement: Supplementary file 1 — Supplementary Figure [file 41598_2018_28865_MOESM1_ESM.docx]

Integrated deep visual and semantic attractor neural networks predict fMRI pattern-information along the ventral object processing pathway

Barry J. Devereux^1^^[[1]](#footnote-1)^, Alex Clarke^1^, & Lorraine K. Tyler^1^ *

Department of Psychology, University of Cambridge, Downing Street, Cambridge, CB2 3EB, United Kingdom.

* Corresponding Author. lktyler@csl.psychol.cam.ac.uk

**Supplementary Figure**


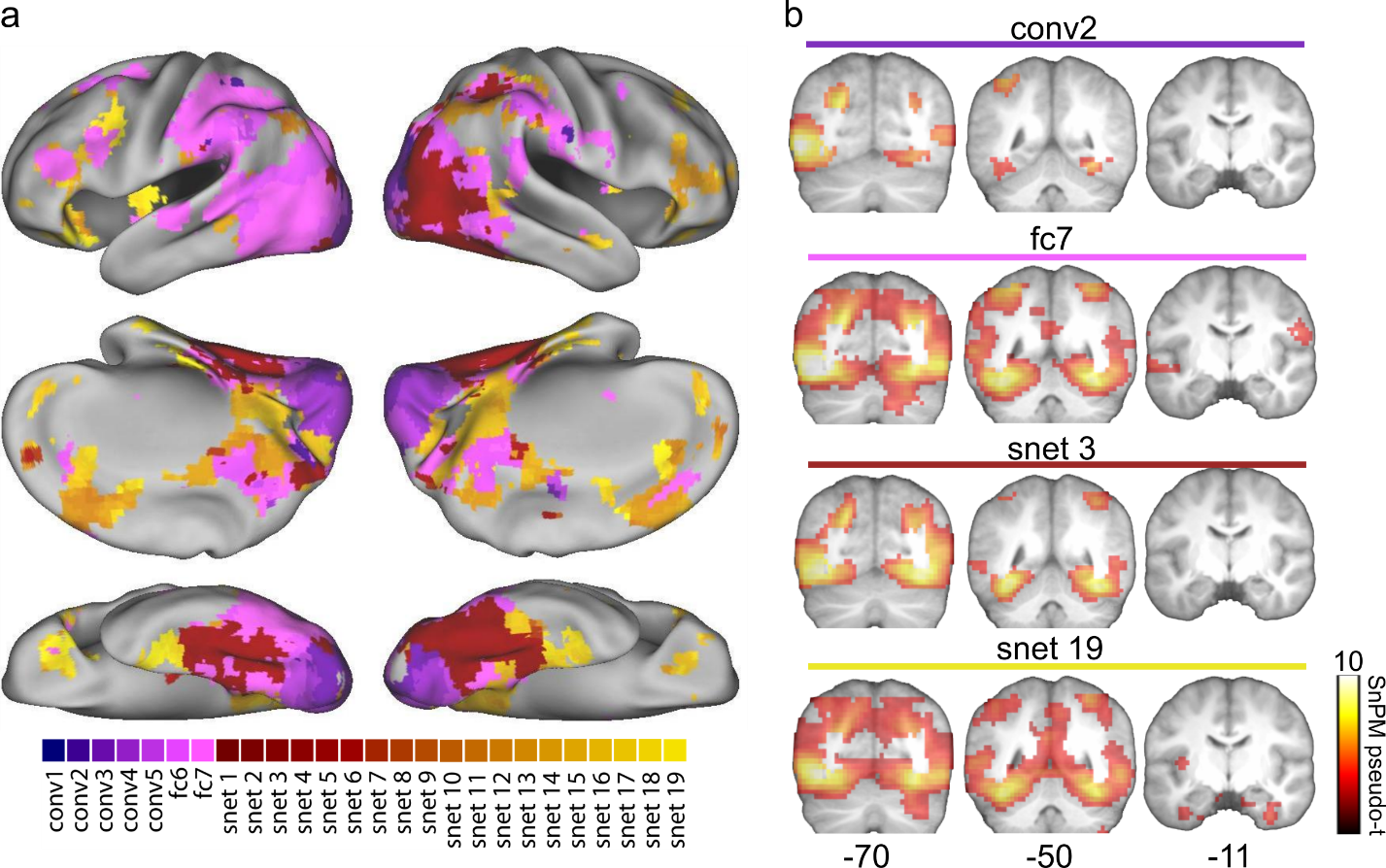


Supp figure 1. Searchlight results comparing representations at each stage of the VS model to representations across the cortex. (a) Composite map of the searchlight results, including a medial view, for all 26 model RDMs. Colors represent the best fitting model RDMs across the cortex. (b) Individual RSA results for 4 stages of the visual+semantic model (same as in Figure 7) at slices covering approximately early visual regions, posterior ventral temporal regions and anterior temporal regions.

1. Present Address: Institute of Electronics, Communications & Information Technology, Queen’s University, Belfast, UK. [↑](#footnote-ref-1)
